# High-energy diets with reduced digestible lysine for Japanese laying quails under thermoneutral conditions: effects on laying performance, egg quality, and profitability

**DOI:** 10.1007/s11250-026-05412-z

**Published:** 2026-10-18

**Authors:** Ana Paula S. Ton, Lidiane Staub, Aldemar M. de Jesus, Anderson Corassa, Adedoyin T. Amos, Adriana G. do Amaral, Ednaldo A. de Andrade, Leonardo W. de Freitas, Maicon Sbardella

**Affiliations:** 1 Núcleo de Estudos em Peixes, Aves e Suínos (NEPAS), Federal University of Mato Grosso (UFMT), Sinop, Mato Grosso 78550-728 Brazil; 2 Department of Animal Nutrition, Federal University of Agriculture, Abeokuta, Ogun State 110124 Nigeria

**Keywords:** Amino acid, Dietary energy, Egg production, Egg quality, Japanese quail, Thermoneutral environment

## Abstract

Japanese quails (*Coturnix coturnix japonica*) are widely used for commercial egg production, requiring an adequate dietary energy-to-amino acid ratio to support egg production and economic returns. However, current recommendations for laying Japanese quails remain inconsistent due to variations among strains, production phases, and environmental conditions. In this study, the interactive effects between dietary metabolizable energy (ME) and digestible lysine (DL) on laying performance, egg quality, and profitability in quails reared in a climate-controlled environment were assessed. Birds were fed diets formulated to contain three ME levels (2600, 2875, or 3150 kcal/kg) combined with four DL levels (0.90, 1.05, 1.20, or 1.35%) in a 3 × 4 factorial arrangement. Responses were monitored over a 32-week experimental period divided into two consecutive 16-week phases (Phase I: 60 to 172 days; Phase II: 173 to 285 days of age). Compared with lower-ME diets, increasing dietary ME to 3150 kcal/kg improved the laying percentage, egg mass, feed-to-egg ratio, and profitability (*P* < 0.05). Conversely, increasing dietary DL above the lowest level tested (0.90%) provided no additional productive or economic benefits, despite ME-dependent interactions affecting eggshell traits and body weight. These findings suggest that, in climate-controlled production systems, Japanese laying quails may benefit from greater dietary energy density than some current recommendations indicate, whereas additional DL supplementation above 0.90% was not associated with consistent improvements in laying performance and profitability. Therefore, high-energy diets formulated with 3150 kcal/kg ME combined with 0.90% DL supported egg production and egg quality while improving feed efficiency and profitability under thermoneutral conditions.

## Introduction

Poultry eggs are an affordable source of animal protein for human nutrition worldwide (Habibi et al. 2024). Among egg-producing species, Japanese quails (*Coturnix coturnix japonica*) offer competitive farming advantages, including low production costs, short generation intervals, early sexual maturity, small body size, disease resistance, and high egg production (Hrncar et al., 2014). However, defining dietary metabolizable energy and digestible lysine levels for Japanese laying quails remains challenging because nutrient responses may vary with genetic strain, husbandry practices, and environmental conditions.

Dietary energy density and the balance between energy and essential amino acid supply are critical in egg-laying poultry because they influence nutrient utilization and productive output (Garcia et al. 2005; Lima et al. 2016; Nahavandinejad et al. 2024). Lysine is a key amino acid in corn- and soybean meal-based diets, with direct implications for egg mass, protein deposition, and eggshell quality (Lima et al. 2016; Cavalcante et al. 2020). Nutritional recommendations and feeding standards for Japanese laying quails commonly indicate dietary ME values around 2800 kcal/kg (NRC, 1994; Rostagno et al. 2011; 2017; 2024) and digestible lysine levels ranging from 1.00 to 1.15% (Costa et al. 2008; Rostagno et al. 2011, 2017, 2024), depending on laying phase, body weight, egg mass, dietary crude protein, lysine-to-amino acid ratio, and the feedstuff composition database adopted. However, previous studies have investigated dietary ME levels ranging from 2500 to 3150 kcal/kg, combined with crude protein levels of 18–22% or digestible lysine levels of 0.90–1.35% (e.g., Fonseca et al. 2021; Ashour et al. 2024; Mariot et al. 2024). More recently, comparisons of nutritional programs based on different feeding guidelines have also reported differences in laying performance in Japanese quails (Lima et al. 2026). Together, these findings highlight the importance of considering dietary formulation, genetic background, and environmental conditions when evaluating nutritional strategies.

In Japanese quails reared in naturally ventilated housing under hot tropical conditions, Fonseca et al. (2021) reported that a diet formulated with 3150 kcal/kg of metabolizable energy combined with 0.90% digestible lysine supported laying percentage, egg mass, and shell quality. In addition, thermoneutral rearing conditions have been shown to mitigate physiological responses to heat stress and improve laying performance and egg quality (El-Tarabany 2016; Wasti et al. 2020; Matos Júnior et al., 2023, 2024; Souza et al. 2025). Thus, transitioning toward climate-controlled housing systems requires re-evaluating nutritional strategies that balance dietary energy and amino acid supply, thereby providing a practical approach to optimizing performance over prolonged production cycles.

In this study, the interactive effects of dietary metabolizable energy (ME) and digestible lysine (DL) on the laying performance, egg quality, and profitability of Japanese quails reared under thermoneutral conditions were evaluated. Birds were fed diets formulated with three ME levels (2600, 2875, or 3150 kcal/kg) combined with four DL levels (0.90, 1.05, 1.20, or 1.35%) over a 32-week feeding period, from 60 to 285 days of age. These levels were selected to encompass ME and DL concentrations below, within, and above commonly recommended values.

## Materials and methods

The experiment was conducted from July 2015 to February 2016 at the Center for Monogastric Nutrition Experimentation of the Institute of Agrarian and Environmental Sciences, Federal University of Mato Grosso (UFMT), in Sinop, Mato Grosso, Brazil. The experimental procedures and dietary matrix were developed within the same long-term Japanese quail nutrition research program described by Fonseca et al. (2021); however, the present study evaluated Japanese laying quails under climate-controlled thermoneutral conditions rather than the non-climate-controlled tropical conditions used in that study. Part of the data reported in this manuscript was generated within the Master’s dissertations of Lidiane Staub and Aldemar M. de Jesus at UFMT (Staub 2016; Marques de Jesus 2018).

### Animals, experimental design, treatments and diets

Five hundred forty female Japanese quails of the Fujikura strain (*Coturnix coturnix japonica*), weighing 145 ± 2 g, were allotted to 12 dietary treatments in a completely randomized design with a 3 × 4 factorial arrangement of treatments, with five replicates per treatment and nine quails per experimental unit. The birds were housed in galvanized wire laying cages without nests (25 cm wide × 35 cm deep × 18 cm high; stocking density of 1.03 birds per 100 cm²) equipped with a cup-type drinker, a gutter-type feeder, and an excreta collection tray. The quails were maintained in an air-conditioned climate-controlled room with a 17 h photoperiod using natural plus artificial lighting. Room temperature and relative humidity were recorded hourly throughout the trial using a digital data logger placed at bird height. The average temperature and relative humidity during the experimental period were 22.37 ± 3.66 °C and 66.86 ± 6.53%, respectively. The air-conditioning system was managed to maintain the birds within the thermoneutral zone, according to Silva et al. (2017) and Matos Junior et al. (2023, 2024). All birds had free access to water and mash feed throughout the 32-week feeding period, from 60 to 285 days of age. The feeding period was divided into two physiologically distinct 16-week production phases: Phase I, corresponding to early/peak laying, from 60 to 172 days of age, and Phase II, corresponding to later laying, from 173 to 285 days of age. Each 16-week production phase comprised four 4-week evaluation periods.

The dietary treatments (Table 1) were formulated by combining three ME levels (2600, 2875, or 3150 kcal/kg) with four standardized ileal DL levels (0.90, 1.05, 1.20, or 1.35%). These levels were selected to represent a broad formulation matrix for Japanese laying quails, encompassing dietary energy and digestible lysine values below, within, and above those commonly reported in nutritional recommendations, previous experimental studies, and practical formulation scenarios. The dietary energy gradient was obtained by modifying the inclusion of fibrous ingredients and lipid sources, particularly soybean hulls and soybean oil, while maintaining similar calculated crude protein levels across diets. Crystalline L-lysine•HCl, DL-methionine, and L-threonine were added to adjust the DL gradient and to ensure that the target methionine-plus-cystine-to-lysine and threonine-to-lysine ratios were met or exceeded. Diets were formulated using SuperCRAC^®^ software (TD Sistemas, Viçosa, MG, Brazil), a least-cost linear programming system, based on the feedstuff composition and nutrient requirement tables published by Rostagno et al. (2011). Accordingly, the nutrient composition in Table 1 represents calculated values formulated according to the nutritional matrices database.

**Table 1:** Composition of experimental diets formulated with different levels of metabolizable energy (ME) and digestible lysine (DL)

Metabolizable energy	2600 kcal/kg					2875 kcal/kg					3150 kcal/kg			
Digestible lysine	0.90%	1.05%	1.20%	1.35%		0.90%	1.05%	1.20%	1.35%		0.90%	1.05%	1.20%	1.35%
Ingredients (%):														
Corn	55.6	55.4	55.3	55.3		58.8	59.5	60.2	60.9		52.4	53.0	53.6	54.4
Soybean meal (45% CP)	28.7	27.9	27.0	26.0		30.3	29.5	28.7	27.7		31.4	30.7	29.9	28.9
Soybean hulls	7.10	7.65	8.20	8.75		0.00	0.00	0.00	0.00		0.00	0.00	0.00	0.00
Soybean oil	0.00	0.00	0.00	0.00		2.15	1.90	1.65	1.40		7.45	7.21	6.97	6.73
Dicalcium phosphate	1.08	1.09	1.10	1.10		1.09	1.10	1.11	1.12		1.10	1.11	1.11	1.12
Limestone	6.60	6.60	6.59	6.59		6.68	6.68	6.67	6.68		6.67	6.67	6.67	6.67
DL-Methionine (98%)	0.24	0.37	0.50	0.65		0.23	0.36	0.49	0.63		0.24	0.36	0.49	0.63
L-Lysine•HCl (78%)	0.02	0.24	0.46	0.68		0.01	0.23	0.45	0.67		0.00	0.20	0.42	0.65
L-Threonine (98.5%)	0.00	0.02	0.13	0.24		0.00	0.01	0.11	0.21		0.00	0.00	0.11	0.21
Sodium chloride	0.33	0.33	0.33	0.33		0.32	0.32	0.32	0.32		0.33	0.33	0.33	0.33
Premix^1^	0.40	0.40	0.40	0.40		0.40	0.40	0.40	0.40		0.40	0.40	0.40	0.40
Total	100.00	100.00	100.00	100.00		100.00	100.00	100.00	100.00		100.00	100.00	100.00	100.00
Calculated nutrient composition:^2^														
Metabolizable energy (kcal/kg)	2600	2600	2600	2600		2875	2875	2875	2875		3150	3150	3150	3150
Digestible lysine (%)	0.90	1.05	1.20	1.35		0.90	1.05	1.20	1.35		0.90	1.05	1.20	1.35
Crude protein (%)	18.49	18.50	18.51	18.50		18.51	18.49	18.50	18.52		18.50	18.50	18.49	18.50
Digestible methionine (%)	0.49	0.61	0.73	0.87		0.48	0.60	0.72	0.86		0.48	0.60	0.73	0.86
Digestible met + cyst (%)	0.74	0.86	0.98	1.11		0.74	0.86	0.98	1.11		0.74	0.86	0.98	1.11
Digestible threonine (%)	0.62	0.63	0.72	0.81		0.64	0.63	0.72	0.81		0.64	0.63	0.72	0.81
Digestible tryptophan (%)	0.20	0.19	0.19	0.18		0.21	0.20	0.20	0.19		0.21	0.20	0.20	0.20
Digestible valine (%)	0.78	0.76	0.75	0.73		0.79	0.78	0.76	0.75		0.79	0.78	0.77	0.75
Digestible histidine (%)	0.45	0.44	0.43	0.42		0.46	0.46	0.45	0.44		0.46	0.46	0.45	0.44
Digestible isoleucine (%)	0.71	0.69	0.68	0.66		0.72	0.71	0.70	0.68		0.73	0.72	0.70	0.69
Digestible leucine (%)	1.46	1.43	1.41	1.38		1.50	1.48	1.46	1.43		1.48	1.46	1.44	1.41
Digestible arginine (%)	1.15	1.12	1.10	1.07		1.16	1.14	1.11	1.09		1.18	1.15	1.13	1.10
Digestible phenylalanine (%)	0.84	0.83	0.81	0.79		0.86	0.85	0.83	0.81		0.86	0.85	0.83	0.82
Digestible phe + tyr (%)	1.44	1.41	1.38	1.35		1.47	1.45	1.42	1.39		1.48	1.45	1.43	1.40
Ether extract (%)	2.73	2.73	2.72	2.72		4.80	4.56	4.33	4.09		9.86	9.64	9.41	9.18
Linoleic acid (%)	1.43	1.43	1.43	1.43		2.52	2.40	2.27	2.15		5.20	5.07	4.95	4.83
Calcium (%)	2.92	2.92	2.92	2.92		2.92	2.92	2.92	2.92		2.92	2.92	2.92	2.92
Available phosphorus (%)	0.30	0.30	0.30	0.30		0.30	0.30	0.30	0.30		0.30	0.30	0.30	0.30
Sodium (%)	0.15	0.15	0.15	0.15		0.15	0.15	0.15	0.15		0.15	0.15	0.15	0.15
Crude fiber (%)	4.82	4.94	5.07	5.20		2.63	2.60	2.56	2.53		2.58	2.55	2.51	2.48

^1^Trace mineral and vitamin premix guaranteed levels per kg of premix: vitamin A, 2,700,000 IU; vitamin D₃, 1,000,000 IU; vitamin E, 2,000 mg; thiamine, 350 mg; riboflavin, 1,000 mg; pyridoxine, 600 mg; vitamin B₁₂, 3,000 µg; vitamin K₃, 400 mg; folic acid, 100 mg; biotin, 10 mg; calcium pantothenate, 2,200 mg; niacin, 6,000 mg; choline chloride, 26 g; zinc oxide, 30 g; iron sulfate, 18 g; manganese oxide, 35 g; copper sulfate, 2,700 mg; cobalt sulfate, 70 mg; calcium iodide, 260 mg; sodium selenite, 100 mg; antioxidant, 1,200 mg; and ethoxyquin, 500 mg

^2^Calculated nutrient composition based on the feedstuff composition tables of Rostagno et al. (2011)

### Laying performance

Eggs were collected, counted, weighed and classified daily by experimental unit (cage) to determine the egg mass (g/bird/day), laying percentage (%), and marketable eggs (% of laid eggs that were intact and free of visible defects). Body weight and cumulative feed intake were recorded every 4 weeks by experimental unit to calculate the average body weight (g), average daily feed intake (g/bird/day), average daily DL intake (mg/bird/day), and average daily ME intake (kcal/bird/day). Feed intake was corrected for mortality according to Sakomura and Rostagno (2016). The feed-to-egg ratio was calculated using cage-level values and expressed as grams of feed per gram of egg mass (g/g) and kilograms of feed per dozen eggs produced (kg/dz). Additionally, dietary DL and ME efficiencies were calculated at the cage level by dividing total DL and ME intake by total egg mass produced (mg/g and kcal/g, respectively).

### Egg quality

During the final four consecutive days of each 4-week evaluation period, three eggs per replicate cage were collected based on proximity to the average cage-level egg weight (± 10%), resulting in a total of 60 sampled eggs per treatment per evaluation period. All the sampled eggs were used for the full set of internal and external egg quality analyses, including the following parameters: specific gravity (g/cm³), according to Castelló et al. (1989); Haugh unit, according to Stadelman et al. (1995); shell thickness (mm), according to Silva and Santos (2000); shell, albumen, and yolk percentages (%), calculated relative to whole-egg weight; albumen pH, measured using a PHS-3B pH meter with a probe; and yolk color, determined using a Minolta CR-400 colorimeter in the CIELab system, following Bible and Singha (1993).

### Economic analyses

Gross income, margin over feed cost, and profitability index were calculated according to Martin et al. (1998) as follows: gross income was determined by multiplying the quantity of eggs produced by the sales price per egg, representing the monetary value obtained from egg sales; margin over feed cost was calculated by subtracting the feeding costs (feed intake multiplied by feed cost) from the gross income; and the profitability index (%) was determined by dividing the margin over feed cost by gross income and multiplying by 100, indicating the percentage of available revenue after the payment of feed costs.

### Statistical analyses

Phase I and Phase II datasets were organized by averaging replicate cage-level data recorded across the four 4-week evaluation periods within each phase. Statistical analyses were performed using SISVAR software (Ferreira 2011). Data were checked for residual normality, homogeneity of variance, and the presence of outliers. Data were subsequently subjected to analysis of variance (ANOVA) according to a completely randomized design with a 3 × 4 factorial arrangement of treatments, using the phase-specific mean of each replicate cage, according to the following model:

Y_ijk_ = µ + ME_i_ + DL_j_ + (ME × DL)_ij_ + e_ijk_,

where Y_ijk_ is the observed value in the k-th replicate cage receiving the i-th level of metabolizable energy (ME) and the j-th level of digestible lysine (DL); µ is the overall mean; ME_i_ is the fixed effect of metabolizable energy (i = 2600, 2875, or 3150 kcal/kg); DL_j_ is the fixed effect of digestible lysine (j = 0.90, 1.05, 1.20, or 1.35%); (ME × DL)_ij_ is the interaction between metabolizable energy and digestible lysine; and e_ijk_ is the random error.

The main-effect means and ANOVA results are presented in the tables. Significant ME × DL interactions (*P* < 0.05) were further explored by linear and quadratic regression analyses of DL within each ME level and are presented graphically. In the absence of significant interactions, the main effects of dietary ME were compared using the Scott–Knott test, whereas the dose‒response effects of dietary DL were evaluated using linear and quadratic regression models.

## Results and discussion

### Body weight and feed and nutrient intake

**Fig. 1:**
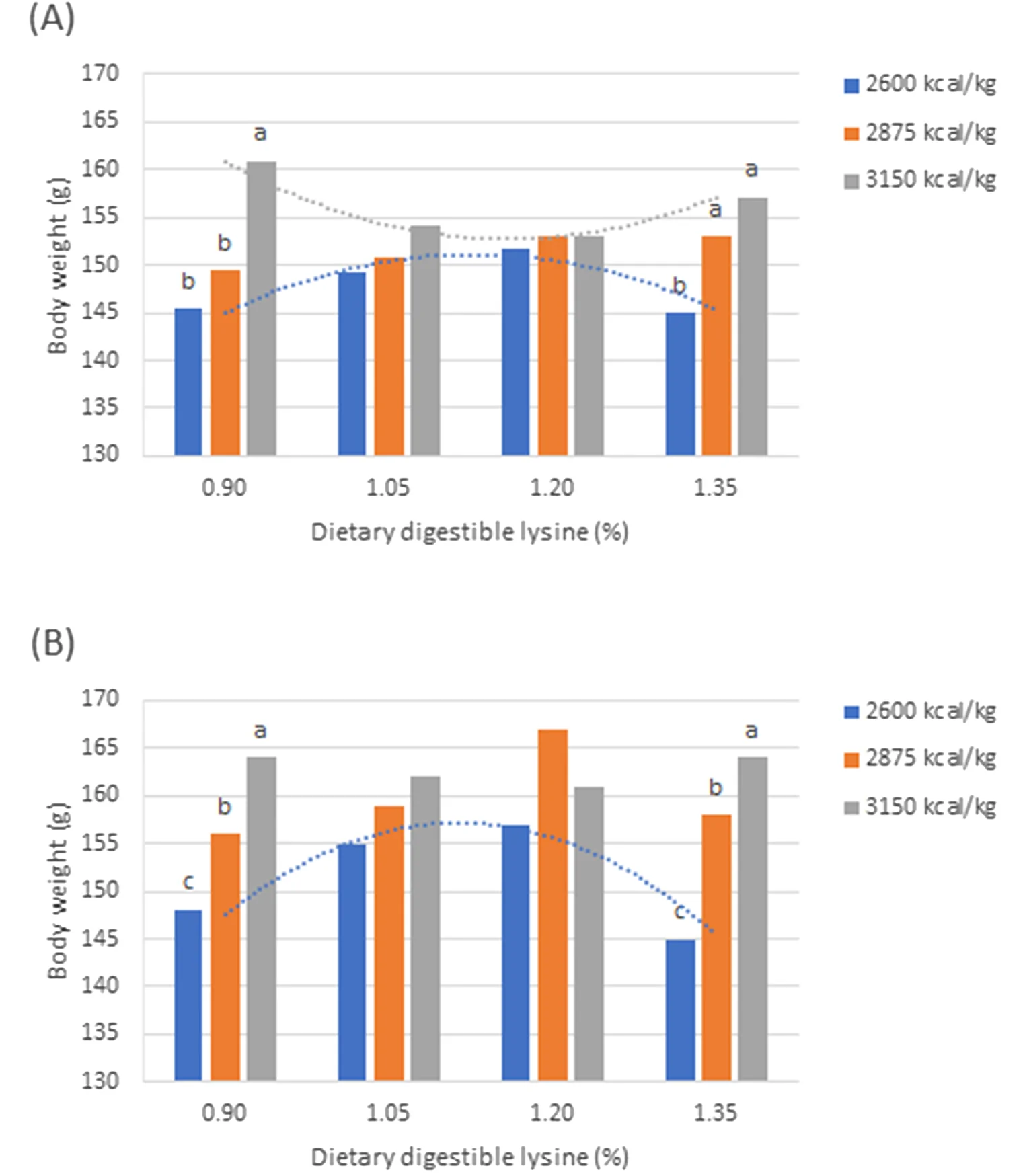
Interactive effects of dietary metabolizable energy (ME) and digestible lysine (DL) on the body weight of Japanese laying quails in Phase I (**A**) and Phase II (**B**) under thermoneutral conditions. Note: Different lowercase letters within the same dietary DL level indicate differences among dietary ME levels according to the Scott–Knott test (*P* < 0.05). (**A**): Quadratic effect of dietary DL at 2600 kcal/kg ME (Y = 0.77 + 266.67 × DL – 118.22 × DL²; R² = 0.91) and at 3150 kcal/kg ME (Y = 314.63–279.28 × DL + 120.44 × DL²; R² = 0.99). (**B**): Quadratic effect of dietary DL at 2600 kcal/kg ME (Y = – 104.75 + 470.33 × DL – 211.11 × DL²; R² = 0.96)

Dietary ME and DL interacted to affect body weight during both production phases (Table 2). Body weight increased with increasing dietary ME level, particularly at the lowest (0.90%) and highest (1.35%) DL levels, whereas intermediate DL levels resulted in higher body weight at 2600 kcal/kg ME (Fig. 1). These results indicate that lower-energy diets, such as those recommended in some feeding standards (< 3000 kcal/kg ME), may have limited body weight accretion during laying, even when DL levels were above 1.05%. Laying birds allocate a substantial proportion of nutrient intake to egg production rather than to body reserve accretion (Mariot et al. 2024). In the present study, egg production traits responded strongly to dietary ME but not to increasing DL levels (Table 3), suggesting that dietary energy was the primary nutritional driver of both productive performance and body weight response under thermoneutral conditions. The ME × DL interaction observed for body weight may also reflect differences in nutrient partitioning between body reserve accretion and egg production, as well as changes in the proportional supply of non-supplemented amino acids relative to lysine at higher DL levels.

**Table 2:** Effects of dietary metabolizable energy (ME) and digestible lysine (DL) levels on the body weight, feed intake, and nutrient intake of Japanese laying quails under thermoneutral conditions

Variable	Dietary ME (kcal/kg)				Dietary DL (%)				CV (%)	*P* value		
	2600	2875	3150		0.90	1.05	1.20	1.35		ME	DL	ME × DL
Phase I (60 to 172 days of age):												
Body weight (g)	147.82	151.58	156.27		151.87	151.42	152.58	151.67	2.83	< 0.001	0.989	0.007
Feed intake (g/bird/day)	22.29	22.59	22.27		22.83	22.19	22.36	22.16	6.04	0.7039	0.5097	0.396
DL intake (mg/bird/day)¹	251.30	252.68	250.52		228.48	242.28	259.93	275.32	5.83	0.6416	< 0.001	0.424
ME intake (kcal/bird/day)	57.96^c^	64.95^b^	70.16^a^		65.78	63.82	64.17	63.67	6.24	< 0.001	0.4623	0.367
Phase II (173 to 285 days of age):												
Body weight (g)	151.25	160.00	162.75		156.00	158.67	161.66	155.67	6.21	< 0.001	0.246	0.001
Feed intake (g/bird/day)	25.08	24.90	25.40		25.07	25.70	25.27	24.47	14.6	0.694	0.346	0.001
DL intake (mg/bird/day)	285.00	282.25	286.00		225.67	270.00	311.33	330.67	15.5	0.854	< 0.001	0.004
ME intake (kcal/bird/day)	65.18	70.98	80.00		72.33	73.57	72.13	70.27	14.3	< 0.001	0.377	0.001

^a−c^ Means followed by different lowercase letters within a row differ among dietary ME levels according to the Scott–Knott test (*P* < 0.05). Dietary DL effects were evaluated by linear and quadratic regression analyses. Significant ME × DL interactions are shown in the corresponding figures

¹Linear effect of dietary DL levels on digestible lysine intake (Y = 132.8 + 105.45 × DL; R^2^ = 0.99)

**Table 3:** Effects of dietary metabolizable energy (ME) and digestible lysine (DL) levels on the egg production and feed efficiency of Japanese laying quails under thermoneutral conditions

Variable	Dietary ME (kcal/kg)				Dietary DL (%)				CV (%)	*P* value		
	2600	2875	3150		0.90	1.05	1.20	1.35		ME	DL	ME × DL
Phase I (60 to 172 days of age):												
Egg mass (g/bird/day)	7.33^c^	8.46^b^	9.00^a^		8.42	8.11	8.43	8.09	10.30	< 0.001	0.553	0.899
Laying percentage (%)	66.73^c^	76.23^b^	80.42^a^		75.72	73.04	75.61	73.47	9.42	< 0.001	0.619	0.966
DL intake per egg (mg/g egg)¹	34.43^c^	30.22^b^	27.93^a^		24.74	29.08	32.20	37.40	7.81	< 0.001	0.001	0.853
Feed-to-egg ratio (g/g)	3.08^c^	2.71^b^	2.50^a^		2.76	2.75	2.78	2.79	8.10	< 0.001	0.545	1.000
Feed-to-egg ratio (kg/dz egg)	0.40^c^	0.36^b^	0.33^a^		0.37	0.37	0.36	0.37	8.09	< 0.001	0.789	0.975
ME intake per egg (kcal/g egg)	7.95^c^	7.82^b^	7.72^a^		7.85	7.82	7.85	7.86	7.58	0.046	0.610	0.997
Marketable eggs (%)	98.10	98.31	98.32		98.48	97.82	98.27	98.40	1.06	0.7555	0.316	0.776
Phase II (173 to 285 days of age):												
Egg mass (g/bird/day)	7.50^c^	8.52^b^	9.36^a^		8.31	8.43	8.50	8.71	24.3	< 0.001	0.073	0.082
Laying percentage (%)	65.10^c^	73.30^b^	79.50^a^		71.80	72.30	74.10	74.20	22.12	< 0.001	0.322	0.072
DL intake per egg (mg/g egg)	33.32	34.64	35.13		33.88	33.97	34.16	35.55	22.45	0.792	0.484	0.091
Feed-to-egg ratio (g/g)	3.31^c^	3.02^b^	2.78^a^		3.17	3.05	3.00	3.17	27.52	< 0.001	0.068	0.069
Feed-to-egg ratio (kg/dz egg)	0.48^c^	0.42^b^	0.39^a^		0.44	0.43	0.41	0.47	25.83	< 0.001	0.326	0.105
ME intake per egg (kcal/g egg)	8.89^c^	8.77^b^	8.61^a^		8.80	8.83	8.79	8.71	26.21	0.042	0.081	0.079
Marketable eggs (%)	97.82	97.90	98.11		97.91	98.22	97.90	97.71	1.81	0.656	0.474	0.161

^a−c^ Means followed by different lowercase letters within a row differ among dietary ME levels according to the Scott–Knott test (*P* < 0.05). Dietary DL effects were evaluated by linear and quadratic regression analyses. Significant ME × DL interactions are shown in the corresponding figures

¹Linear effect of dietary DL levels on DL intake per gram of egg produced (Y = 0.03 + 27.40 × DL; R^2^ = 0.99)

**Fig. 2:**
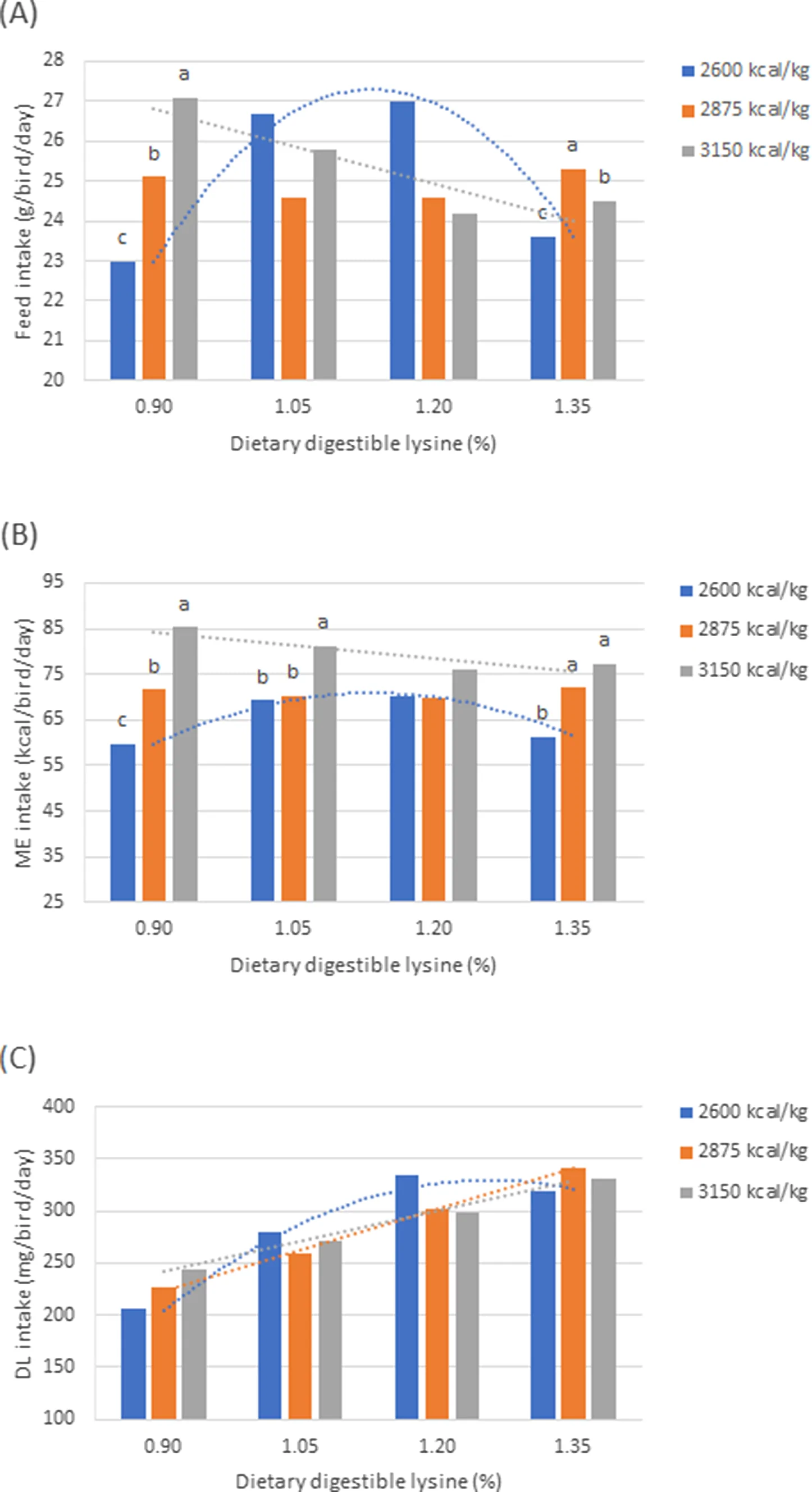
Interactive effects of dietary metabolizable energy (ME) and digestible lysine (DL) on the feed intake (**A**), metabolizable energy intake (**B**) and digestible lysine intake (**C**) of Japanese laying quails in Phase II under thermoneutral conditions. Note:Different lowercase letters within the same dietary DL level indicate differences among dietary ME levels according to the Scott–Knott test (*P* < 0.05). (**A**): Quadratic effect of dietary DL at 2600 kcal/kg ME (Y = – 74.13 + 178.90 × DL – 78.89 × DL²; R² = 0.99) and linear effect at 3150 kcal/kg ME (Y = 32.45–6.27 × DL; R² = 0.83). (**B**): Quadratic effect of dietary DL at 2600 kcal/kg ME (Y = – 190.73 + 461.30 × DL – 203.33 × DL²; R² = 0.99) and linear effect at 3150 kcal/kg ME (Y = 101.75–19.33 × DL; R² = 0.82). (**C**): Quadratic effect of dietary DL at 2600 kcal/kg ME (Y = – 1217.50 + 2460.00 × DL – 977.78 × DL²; R² = 0.99) and linear effects at 2875 kcal/kg ME (Y = – 11.00 + 260.67 × DL; R² = 1.00) and 3150 kcal/kg ME (Y = 70.00 + 192.00 × DL; R² = 1.00)

Feed intake responses differed across production phases (Table 2). In Phase I, feed intake was not affected by dietary ME or DL (*P* > 0.05), whereas the intake of ME and DL increased (*P* < 0.05) with increasing dietary concentration. In Phase II, ME × DL interactions were observed for feed intake, ME intake, and DL intake (*P* < 0.05; Table 2). At 2600 kcal/kg ME, DL had a quadratic effect on feed intake, with intermediate DL levels resulting in greater intake. Conversely, at 3150 kcal/kg ME, increasing DL reduced feed intake linearly (Fig. 2A), with a similar pattern observed for ME intake (Fig. 2B). Digestible lysine intake showed a quadratic response at 2600 kcal/kg ME and linear increases at 2875 and 3150 kcal/kg ME (Fig. 2C). These responses suggest that the effect of DL on voluntary feed intake depends on dietary energy density, reinforcing the need to evaluate energy and amino acid supply together rather than as independent nutritional factors. This interpretation corroborates the findings of earlier studies showing that dietary lysine responses in laying quails depend on the nutritional context of the diet (e.g., Costa et al. 2008; (Morais et al. 2023); Mauricio et al., 2018).

Overall, increasing dietary energy density increased ME intake and body weight without reducing feed intake, while DL concentrations above 0.90% exerted negligible influence on body accretion. This response differs from the conventional expectation that poultry reduce feed intake as dietary energy density increases, as reported in previous studies (e.g., Gheisari et al. 2011; Ashour et al. 2024). However, under thermoneutral conditions, voluntary feed intake in laying Japanese quails may be influenced not only by dietary energy density, but also by diet composition, physical gut fill capacity, and temperature-dependent physiological mechanisms of intake regulation and nutrient partitioning. As a result, the birds maintained relatively stable feed intake (~ 22 g/bird/day in Phase I and ~ 25 g/bird/day in Phase II) across dietary ME levels. This pattern increased daily ME intake as dietary energy density increased (from 57.96 to 70.16 kcal/bird/day in Phase I and from 65.18 to 80.00 kcal/bird/day in Phase II). Therefore, the lower ME values commonly used for laying Japanese quails may not fully support the maintenance of modern strains in climate-controlled systems, especially during long production cycles. This interpretation is consistent with previous evidence of lower feed intake for Japanese quails reared under hot climate conditions in naturally ventilated systems (Fonseca et al. 2021).

### Laying performance

No ME × DL interaction was observed for laying performance (Table 3), although some Phase II variables showed near-threshold interaction probabilities (0.069 ≤ *P* ≤ 0.091) and higher coefficients of variation, suggesting greater heterogeneity among experimental units during the later production phase. This variability was more evident in low-ME diets and appeared to be accentuated at the extreme DL levels, indicating that responses became less uniform when dietary energy supply was limiting. In addition, as no other relevant events, such as abnormal mortality, health problems, equipment failures, or data-entry errors, were identified, this greater variability was more likely associated with biological heterogeneity among birds as laying progressed, including differences in persistency of lay, frequency of non-laying days, and body reserve accumulation.

Considering the main effects, increasing dietary ME improved (*P* < 0.05) egg mass, laying percentage, and the feed-to-egg ratio, thereby reducing ME and DL expenditure per gram of egg produced (Table 3). Notably, higher dietary energy increased the laying percentage from 66.73% to 80.42% in Phase I and from 65.10% to 79.50% in Phase II (Table 3). In Phase I, increasing dietary DL increased DL expenditure per gram of egg produced without increasing laying percentage or egg mass, indicating that DL levels above the lowest level tested (0.90%) were not converted into additional egg output. Egg marketability was not affected (*P* > 0.05) by either ME or DL.

The positive response to dietary ME is consistent with evidence from laying hens, in which increasing dietary energy density can improve laying percentage, egg mass, and feed efficiency (e.g., Li et al. 2013; Ribeiro et al. 2014). A similar response was observed in the present study with Japanese quails, as higher ME improved egg output and feed-to-egg ratio while reducing nutrient expenditure per gram of egg produced. Improved feed conversion with increasing dietary ME has been reported in growing quails (Muniz et al. 2016; Gharanjik et al. 2024). In laying Japanese quails, Ashour et al. (2024) reported increased egg weight and egg mass at higher dietary ME levels, whereas Mariot et al. (2024) observed reduced feed intake without significant improvements in laying performance. Comparable to the present study, Fonseca et al. (2021), using the same dietary ME and DL levels under hot tropical conditions, reported similar laying performance and egg mass at 2,875 and 3,150 kcal/kg. In contrast, both variables increased progressively with dietary ME in the present study under thermoneutral conditions. These contrasting responses suggest that environmental and experimental conditions may influence the productive response to dietary energy, considering the documented effects of ambient temperature on laying performance in Japanese quails (e.g., Souza et al. 2025). Greater metabolizable energy availability may also support ovarian activity, vitellogenesis, and follicular maturation (Lotfi et al. 2018). However, because the ME gradient was obtained by reducing soybean hulls and increasing soybean oil inclusion (Table 1), the observed responses cannot be attributed exclusively to dietary energy density. Associated diet-matrix effects, including lower fiber content, higher lipid and linoleic acid supply, and potential changes in feed intake regulation and nutrient digestion and absorption, may also have contributed. Indeed, studies in laying quails have shown that both the inclusion of alternative feed ingredients (e.g., Dalle Zotte et al. 2026) and the use of different lipid sources in isoenergetic diets (e.g., Valentim et al. 2025) can affect nutrient digestibility and egg production.

Unlike dietary ME, increasing dietary DL above the lowest level tested (0.90%) did not increase the laying percentage or egg mass, but increased DL expenditure per gram of egg produced. This interpretation is consistent with the tendency of Brazilian feeding tables to reduce DL recommendations for Japanese laying quails from earlier to more recent editions, from 1.04 to 1.15% in 2011/2017 to 0.99–1.05% in 2024, although these recommendations are still generally associated with dietary ME levels around 2800–2850 kcal/kg rather than the high-energy diet evaluated in the present study (Rostagno et al. 2011, 2017, 2024). However, because the diets were formulated to maintain similar crude protein levels, increasing DL through crystalline L-lysine·HCl may have changed the proportional supply of non-supplemented amino acids relative to lysine, particularly at the highest DL levels. Therefore, this response should be interpreted within the context of practical corn–soybean meal-based formulations rather than as a complete ideal-protein titration. Accordingly, under the conditions evaluated, increasing dietary energy density was the primary driver of laying percentage, egg mass, and feed efficiency, whereas additional DL supplementation above the lowest level tested was not converted into economically relevant egg output. These findings corroborate previous evidence showing limited productive response to elevated DL in laying quails (Ribeiro et al. 2013; Mauricio et al., 2018). Taken together, these results indicate that high-energy diets sustained persistent average laying rates (~ 80%) over 32 weeks under thermoneutral housing, without consistent productive benefits from increasing dietary DL above 0.90%.

### Egg quality

**Fig. 3:**
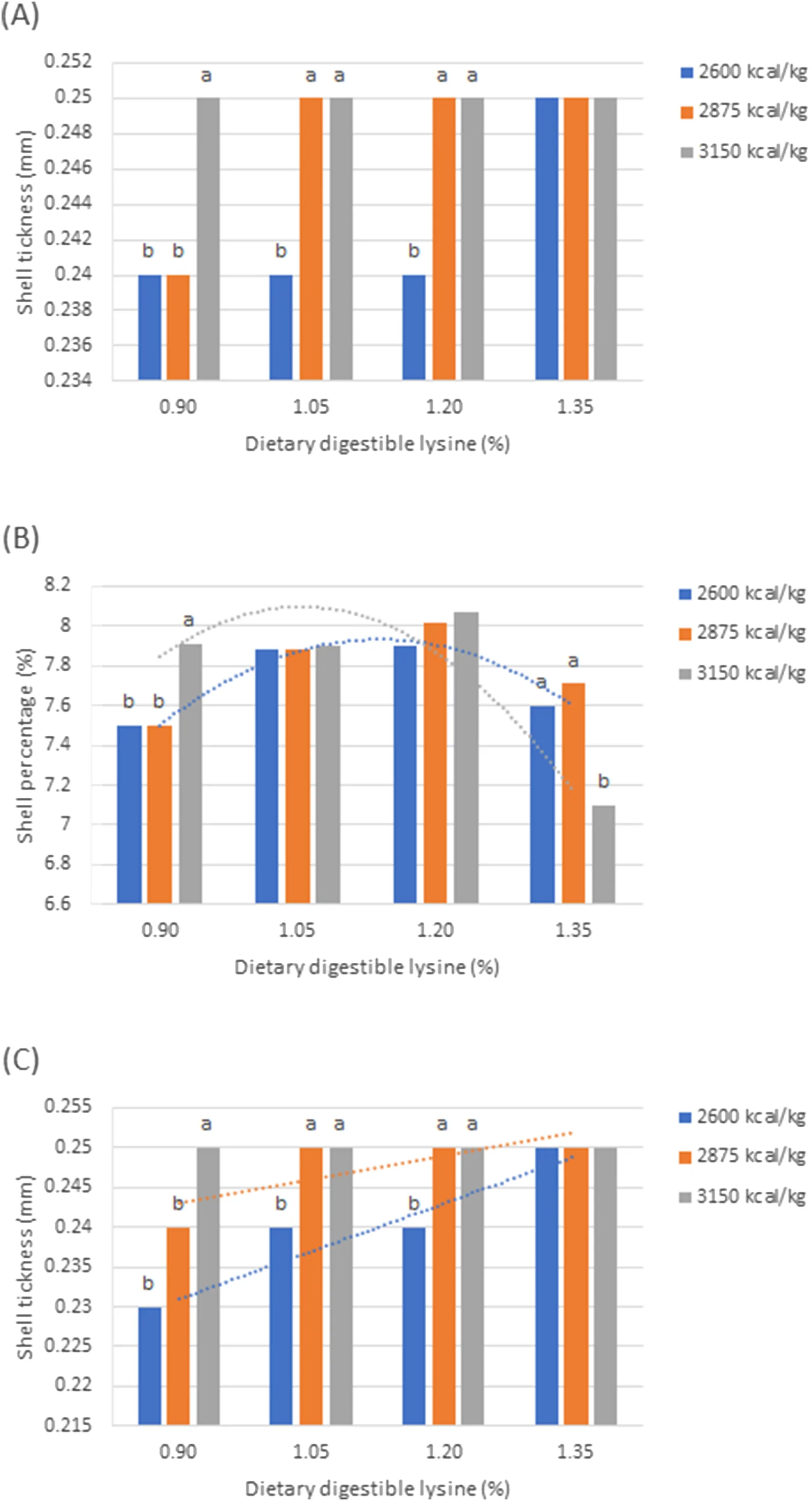
Interactive effects of dietary metabolizable energy (ME) and digestible lysine (DL) on shell thickness in Phase I (**A**), shell percentage in Phase I (**B**), and shell thickness in Phase II (**C**) of Japanese laying quails under thermoneutral conditions. Note: Different lowercase letters within the same dietary DL level indicate differences among dietary ME levels according to the Scott–Knott test (*P* < 0.05). (**A**): No significant linear or quadratic regression effects of dietary DL within ME levels (*P* > 0.05). (**B**): Quadratic effect of dietary DL at 2600 kcal/kg ME (Y = – 1.87 + 17.21 × DL – 7.56 × DL²; R² = 1.00) and at 3150 kcal/kg ME (Y = – 3.76 + 22.49 × DL – 10.67 × DL²; R² = 0.85). (**C**): Linear effect of dietary DL at 2600 kcal/kg ME (Y = 0.195 + 0.040 × DL; R² = 0.90) and at 2875 kcal/kg ME (Y = 0.225 + 0.020 × DL; R² = 0.60)

Dietary ME and DL interacted to affect eggshell traits (Table 4). In Phase I, the interaction affected shell thickness, although no significant linear or quadratic DL response was detected within individual ME levels (Fig. 3A). Shell percentage showed a quadratic response to DL at 2600 and 3150 kcal/kg ME in Phase I (Fig. 3B). In Phase II, shell thickness increased linearly with DL at 2600 and 2875 kcal/kg ME (Fig. 3C). Similarly, Omary et al. (2024) reported quadratic responses in eggshell thickness and relative shell weight to increasing dietary digestible threonine levels in Japanese quail breeders. However, these changes in shell traits did not translate into improvements in marketable egg percentage (Table 3), indicating a limited practical impact.

**Table 4:** Effects of varying dietary levels of metabolizable energy (ME) and digestible lysine (DL) on the egg quality traits of Japanese quails in the laying phase under thermoneutral conditions

Variables	Dietary ME (kcal/kg)				Dietary DL (%)				CV (%)	*P* value		
	2600	2875	3150		0.90	1.05	1.20	1.35		ME	DL	ME × DL
Phase I (60 to 172 days of age):												
Egg weight (g/egg)	11.09	11.29	11.32		11.24	11.26	11.31	11.12	2.33	0.116	0.224	0.117
Albumen percentage (%)	62.35	62.76	62.39		62.43	62.50	62.19	62.88	1.49	0.313	0.254	0.871
Yolk percentage (%)	30.25	29.85	29.88		29.91	30.00	29.96	30.12	1.93	0.062	0.788	0.422
Specific gravity (g/cm³)	1.08	1.08	1.08		1.08	1.08	1.08	1.08	0.25	0.330	0.252	0.418
Albumen pH	8.85	8.84	8.85		8.85	8.86	8.83	8.85	0.45	0.477	0.402	0.520
Albumen height (mm)	4.66	4.68	4.67		4.68	4.70	4.64	4.68	6.17	0.939	0.891	0.834
Haugh unit	92.11	92.04	91.62		91.69	91.91	91.80	92.29	1.04	0.222	0.367	0.221
Yolk yellow coloring (b^*^)	39.57^c^	40.50^b^	41.46^a^		40.95	40.66	40.07	40.36	2.59	0.000	0.134	0.215
Yolk luminosity (L^*^)	54.37	54.23	54.35		54.54	54.17	54.29	54.27	1.23	0.784	0.483	0.336
Shell thickness (mm)	0.24	0.25	0.25		0.24	0.25	0.25	0.25	2.43	< 0.001	0.012	0.003
Shell percentage (%)	7.72	7.75	7.78		7.64	7.89	7.99	7.47	2.91	0.003	< 0.001	0.002
Phase II (173 to 285 days of age):												
Egg weight (g/egg)	11.41	11.61	11.74		11.62	11.67	11.60	11.45	4.10	0.061	0.065	0.500
Albumen percentage (%)	61.01	61.19	61.18		61.49	61.08	60.95	60.99	2.30	0.669	0.136	0.418
Yolk percentage (%)	31.27	30.91	30.92		30.84	31.03	31.06	31.20	4.10	0.134	0.487	0.768
Specific gravity (g/cm³)	1.08	1.08	1.08		1.08	1.08	1.08	1.08	0.34	0.110	0.120	0.056
Albumen pH	8.82	8.79	8.81		8.81	8.80	8.81	8.81	1.19	0.389	0.949	0.165
Albumen height (mm)	4.67	4.69	4.67		4.68	4.70	4.64	4.80	6.17	0.885	0.759	0.464
Haugh unit	90.61	90.54	90.32		90.47	90.56	90.30	90.63	1.72	0.481	0.680	0.414
Yolk yellow coloring (b^*^)	38.80^b^	38.90^b^	39.60^a^		39.20	39.20	38.90	39.30	4.90	0.022	0.621	0.291
Yolk luminosity (L^*^)	55.20	54.80	54.60		54.70	54.80	54.60	55.20	5.50	0.446	0.667	0.220
Shell thickness (mm)	0.25	0.25	0.25		0.24	0.25	0.25	0.25	2.43	0.072	0.207	0.001
Shell percentage (%)	7.73^b^	7.81^b^	7.93^a^		7.73	7.83	7.84	7.88	2.91	0.028	0.321	0.387

^a−c^ Means followed by different lowercase letters within a row differ among dietary ME levels according to the Scott–Knott test (*P* < 0.05). Dietary DL effects were evaluated by linear and quadratic regression analyses. Significant ME × DL interactions are shown in the corresponding figures

The marketable egg output is strongly influenced by environmental conditions. Fonseca et al. (2021) reported that higher dietary ME increased the percentage of marketable eggs in Japanese quails reared under hot climate conditions, whereas no such response was observed in the present study under thermoneutral conditions. This difference suggests that climate-controlled housing may reduce the relative impact of dietary changes on external egg quality and egg marketability. This interpretation is consistent with that of El-Tarabany (2016), who reported that thermal conditions influence both internal and external egg quality.

No ME × DL interaction was detected for the other egg quality traits (Table 4). Dietary DL had no significant main effect, whereas dietary ME increased yolk yellowness (b*). The increase in yolk pigmentation may be associated with the higher dietary fat content of the energy-dense diets (Table 1), which could favor the absorption and deposition of lipophilic carotenoids in the yolk. Similar responses have been reported in quails fed carotenoid-rich or oil-supplemented diets (Santana et al. 2021; Abd El-Hack et al. 2024).

### Profitability

No ME × DL interaction was observed for the economic variables (Table 5). Increasing dietary ME improved (*P* < 0.05) the profitability index, gross income, and margin over the feed cost, whereas dietary DL had no effect on economic outcomes. These improvements were directly associated with greater egg mass, a higher laying percentage, and a better feed-to-egg ratio achieved with higher-ME diets. Interestingly, in Phase II, despite the greater cost of high-ME diets due to higher soybean oil inclusion (Table 1), the profitability index was greatest at 3150 kcal/kg ME (62.70%; Table 5). This response reflects the higher laying percentage and egg output observed in high-ME diets (Table 3), which diluted feed cost per unit of egg produced. Our findings are consistent with those of Fonseca et al. (2021), who reported that increasing dietary ME enhanced egg output and profitability in Japanese quails reared under hot climate conditions. This long-term evaluation provides practical evidence that dietary energy density is a key factor in cost-effective feed formulation for Japanese quails, whereas formulating with DL above the lowest level tested (0.90%) did not provide a marginal financial return under thermoneutral conditions.

**Table 5:** Effects of dietary metabolizable energy (ME) and digestible lysine (DL) levels on the economic outcomes of Japanese laying quails under thermoneutral conditions

Variable	Dietary ME (kcal/kg)				Dietary DL (%)				CV (%)	*P* value		
	2600	2875	3150		0.90	1.05	1.20	1.35		ME	DL	ME × DL
Phase I (60 to 172 days of age):												
Profitability index (%)	76.78^c^	78.23^b^	79.51^a^		76.28	76.08	76.81	75.58	3.61	0.022	0.066	0.650
Gross income (US$/day)	0.94^b^	1.13^a^	1.15^a^		1.07	1.09	1.09	1.09	16.99	0.026	0.068	0.645
Margin over feed cost (US$/day)	0.71^c^	0.89^b^	0.92^a^		0.81	0.84	0.87	0.87	19.66	0.038	0.062	0.314
Phase II (173 to 285 days of age):												
Profitability index (%)	59.39^b^	60.37^b^	62.70^a^		60.85	60.21	60.79	60.65	15.21	0.026	0.114	0.225
Gross income (US$/day)	0.59^b^	0.70^a^	0.72^a^		0.66	0.67	0.68	0.63	20.72	< 0.001	0.096	0.118
Margin over feed cost (US$/day)	0.36^b^	0.43^a^	0.43^a^		0.42	0.42	0.42	0.38	31.22	< 0.001	0.169	0.236

^a−c^ Means followed by different lowercase letters within a row differ among dietary ME levels according to the Scott–Knott test (*P* < 0.05). Dietary DL effects were evaluated by linear and quadratic regression analyses. Significant ME × DL interactions are shown in the corresponding figures

In applied quail nutrition, however, the economic viability of adopting such high-ME formulations depends heavily on the precision of the raw matrix values. Special attention should be given to the nutrient composition and digestibility values used for feed formulation. In many reference systems, these values are extrapolated from broiler chickens or roosters rather than determined directly in quails (e.g., Rostagno et al. 2011; 2017; 2024). Although quail-specific determinations of metabolizable energy and amino acid digestibility have been generated for some feedstuffs (e.g., Ghazaghi et al. 2022), their applicability may vary across ingredients, strains, ages, and production systems. Therefore, nutritionists should ensure consistency between the feedstuff composition database and the nutritional recommendations adopted to minimize economic risks and fully secure the performance benefits when formulating high-density diets for Japanese laying quails. Recent studies in Japanese quails have demonstrated genetic gains in egg production following selection for early laying (e.g., Manaa et al. 2024) and differences in productive performance and egg quality among nutritional programs (e.g., Lima et al. 2026). Whether such genetic gains also modify daily energy requirements, particularly over prolonged laying cycles, warrants further investigation. Accordingly, the present findings should be interpreted considering genetic background, body weight, egg output, dietary matrix profile, management practices, housing system, and environmental conditions when applied to other production settings.

## Conclusions

High-energy diets formulated with 3150 kcal/kg ME and the lowest DL level tested (0.90%) improved laying performance, egg mass, feed efficiency, and profitability of Japanese quails maintained under thermoneutral conditions over a 32-week production cycle. From a practical farming perspective, increasing dietary DL above 0.90% provided no additional benefits under the conditions evaluated, despite ME-dependent interactions affecting eggshell traits and body weight. The findings from this long-term evaluation indicate that higher dietary energy density combined with reduced digestible lysine may represent a cost-effective feed formulation approach for Japanese laying quails of similar genetic background fed corn-soybean meal-based diets and maintained in climate-controlled tropical systems.

## Data Availability

The datasets generated during and/or analyzed during the current study are available upon request from the corresponding author.
